# Validity and reliability of the Persian version of the nurse spiritual care therapeutics scale (NSCTS)

**DOI:** 10.1186/s12904-021-00750-1

**Published:** 2021-04-12

**Authors:** Fatemeh Merati-Fashi, Behnam Khaledi-Paveh, Hadis Mosafer, Abbas Ebadi

**Affiliations:** 1 Department of Nursing, Faculty of Nursing and Midwifery, Tehran Medical Sciences, Islamic Azad University, Tehran, Iran; 2 Sleep Disorders Research Center, Kermanshah University of Medical Sciences, Kermanshah, Iran; 3 Department of Psychiatric Nursing, Scholl of Nursing and Midwifery, Kermanshah University of Medical Sciences, Kermanshah, Iran; 4 Nursing Department, Student Research Committee, Nursing and Midwifery School, Kermanshah University of Medical Sciences, Kermanshah, Iran; 5 Behavioral Sciences Research Center, Life style institute, Baqiyatallah University of Medical Sciences, Tehran, IR Iran; 6 Nursing Faculty, Baqiyatallah University of Medical Sciences, Tehran, IR Iran

**Keywords:** Spiritual care, Nurse, Validity, Reliability, Persian

## Abstract

**Background:**

Dealing with the spiritual needs of patients has been recognized as one of the principles of holistic care in nursing. Therefore, it is necessary for nurses to deal with the spiritual issues of patients. Also, a valid and reliable scale is needed to measure nurse-provided spiritual care. So the purpose of this study was to determine the validity and reliability of the Persian version of the “Nurse Spiritual Care Therapeutics Scale” in Iranian nurses.

**Method:**

In a methodological study, English version of the Nurse Spiritual Care Therapeutics Scale was translated into Persian by forward–backward translation procedure. Face validity was assessed by cognitive interview, and content validity was assessed by a panel of experts. In addition, construct validity was assessed by exploratory factor analysis. The participants were 188 nurses working in different adult wards. Reliability was measured using the Cronbach’s alpha and stability reliability was assessed using the internal correlation coefficient (ICC).

**Results:**

In assessing the construct validity, two factors with eigenvalues greater than 1 were identified, which explained 58.47% of the total variance. Cronbach’s alpha value was 0.932, and ICC was 0.892.

**Conclusion:**

As a result, the Persian version of the Nurse Spiritual Care Therapeutics Scale shows a good validity and reliability. Therefore, this scale can be used to evaluate spiritual care at the bedside in Iran.

## Introduction

Spiritual care is a unique aspect of comprehensive nursing care that cannot be replaced by social, psychological, or religious care [1]. There is a complex relationship between patient and nurse to promote patients’ spirituality [2]. This concept is multidimensional and so important that NHS Education for Scotland (NES) defines spiritual care as the care that a nurse recognizes and responds to the needs of the human spirit when faced with trauma, illness or grief [3], and includes activities such as respect and maintaining the patient’s spirituality and dignity, listening carefully to the patients, and helping them to understand the meaning of their illness by nurses [4]. Spiritual care by nurses has positive effects such as inner peace, satisfaction, appreciation, comfort, self-awareness, more ability to cope, reducing pain, improving self-management in type 2 diabetic patients, and increasing spiritual awareness and satisfaction in the population of elderly patients and chronic patients who require long-term care [5].

Although the concept of spiritual care is deeply embedded in the field of nursing, and despite the fact that in the recent decade at least 20 nursing books on spiritual care have been published, and many nursing texts include chapters on spirituality with spiritual care implications, but a review of the literature indicates that spiritual care is not a work priority for nurses [6] and they do not assess the patient’s spiritual needs. For example, in Martins’s (2013) study which was done in Portugal, it was revealed that despite the fact that nurses acknowledge their role in spirituality and spiritual care, but they consider it very abstract and do not pay much attention to it and do not do spiritual care [7]. Besides, studies in Turkey have shown that the level of awareness on the subject was not enough, the spiritual needs of patients were ignored, and nurses tended to regard the spirit as a religious imperative. In these studies, 50.7% of nurses performed spiritual care. The spiritual needs of most patients were not assess and 88.8% of nurses did not provide spiritual interventions. About this, 46.9% of nurses said they did not know what kind of intervention they should provide and 45.2% said spiritual care was not their duty. Therefore, according to the obtained results, the most important reason for not providing spiritual care is lack of awareness and lack of training in this field [8].

Another factor that causes spiritual care to be ignored by nurses is the lack of appropriate tool. Further research shows that most tools measure a nurse’s attitude, beliefs, or perceptions about spiritual care. For example, the tool designed by McSherry, Draper and Kendrick (2002) for assessing spirituality and spiritual care requires nurses to show their satisfaction with spiritual care. In other words, while advances have been made in measuring the structure of spiritual care, most existing tools are multidimensional which confuse spiritual care with attitude and belief [6]. If researchers want to examine the impact of spiritual nursing care interventions in the future, first, there must be an accurate, valid, and reliable tool that evaluates the frequency of this type of care in nursing practice without being affected by the spiritual care provided by the nurse. Also review of literatures has shown that none of the available tools measured the frequency of nurse-provided spiritual care Therefore, the purpose of this study was psychometrics properties and localization of a tool that measures the frequency of spiritual nursing care in Iran. Due to Iran’s Muslim community and different culture from Western countries, there was no scale for evaluating spiritual nursing care [9]. This study is thought to be necessary in order to reveal differences in spiritual understanding. Therefore, the present study was done to answer the research question; how valid and reliable is the Nurse Spiritual Care Therapeutics Scale (NSCTS) in Iran?

## Method

### Ethical considerations

The objectives of the study were explained to the participants and they were assured of the confidentiality of personal information and data obtained. Also, informed written consent was obtained from the participants before starting the study. This study was done in accordance with the Declaration of Helsinki.

#### Participants

The sample of study includes nurses working in different adult wards in the provinces of Tehran, Kermanshah, Hamedan and West Azerbaijan, in the central and western regions of Iran. They were entered into the study by available sampling method based on having at least 1 y of work experience in adult wards. The sample size in this study was 200 people. This is 10 times more than the amount of scale items. The scale was provided to the participants, in-person and electronically. Finally, 188 questionnaires were completed and delivered.

#### Nurse spiritual care therapeutics scale (NSCTS)

The NSCTS is a scale, designed to measure the frequency of nursing activities and specifically to support the patient’s spiritual integrity. Nurses in 12- or 8-h shifts must consider the last 72 or 80 h to respond. It is need to mention that the patient in this scale means any person who received nursing spiritual care (for example, family members and patients).).

This scale contains 17 items that were developed after an extensive review of nursing research identifying spiritual care interventions recognized by nurses. The items in the Likert scale were designed as the number of times spiritual care was performed. These items are as followed: Never (0 times), 1–2 times, 3–6 times, 7–11 times, at least 12 times. The obtained scores were between 17 (minimum) and 85 (maximum). Cronbach’s α coefficient of the scale was 0.93–0.94 and during exploratory factor analysis only one factor extracted that explained 49.5% of the total variance [6].

#### Study design

The present study is a methodological study that was conducted in 2019–2020, in order to localize the Nurse Spiritual Care Therapeutics Scale (NSCTS).

#### Reliability and validity of the tool

##### Translation

According to the standard defined in the Reliability and validity of tools by the World Health Organization (WHO), permission was first obtained from Ms. Elizabeth Johnson at Loma Linda University, the original developer of this scale in English. The scale was translated into Persian by two translators specializing in nursing and fluent in English. Then the translations were studied by a third expert and the best translation was selected. The Persian version of the scale was provided to two other experts and a backward translation was performed. The translated version in English was provided to the developer of the scale and was reviewed by her and points of view were applied in the translation.

##### Face and content validity

Cognitive interview method was used for determining face validity. The Persian version of the scale was provided to 5 nurses working in the hospital. Face validity was performed and due to religious and cultural differences, some words in the scale items were changed. Then content validity was performed using qualitative method and opinions of 5 nursing experts. At this stage, semantic convergence, comprehension, clarity and difficulty of the items were examined.

##### Item analysis

At this stage, the final and modified version of the scale was given to 30 participants. Using SPSS 18 software and loop technique, the correlation between items and the correlation of each item with the total score was measured. Cronbach’s alpha was also examined after the removal of each item.

##### Construct validity

Exploratory factor analysis was used for construct validity. In this method, sampling adequacy was evaluated Kaiser-Meyer-Olkin (KMO) test (KMO > 0.7). KMO measure close to 1 indicates more sampling adequacy for factor analysis, and KMO level between 0.7 and 0.8, and more than 0.8 to 0.9 was considered good. Then the correlation matrix between the variables was evaluated using Bartlett’s Test of Sphericity for factor analysis at an error level of less than 0.05. To extract the factors, the Maximum Likelihood (ML) method and Scree plot were used. Also, the rotation of the factors was done by Promax rotation. In this study the rotation of the factors was measured by Promax method. The reason was due to dependence of the factors on each other; thus the logic of the correlation between items measured better with this method than other rotations. Data analysis was performed by SPSS-18 software.

##### Reliability

Reliability was assessed using two methods of internal consistency including Cronbach’s alpha and test-retest, in two ways; generally and separation of factors. In the test-retest stage, the scale was given to 30 participants at two-week intervals. Then interclass correlation coefficient (ICC) was calculated with a 95% confidence interval, and values above 0.7 were accepted for stability of scale.

## Results

### Samples

The mean age of participants in this study was 31 years, of which 60% were women. Most participants (12.1%) had 1 y of work experience and the mean work experience of participants was 8 years.

### Face and content validity

After reviewing by nursing experts, due to cultural differences, some items were translated in accordance with Iranian culture. For example, item number 15 “offered to read a spiritually nurturing passage (e.g. patient’s Holy Scripture)” was translated as “offered to read spiritually nurturing passage (e.g., the book of the Quran)” and instead of the word “Holy Scripture”, “the book of the Quran” was replaced. There was no cultural difference in other items and they were translated in the same way with the opinion of experts. In content validity, semantic convergence, comprehension, clarity and difficulty of items were assessed. The scale was considered appropriate by content validity experts.

### Item analysis

The correlation between items was 0.4 to 0.8. The highest correlation is related to items 5 and the lowest correlation is related to items 8 and 17. The results are presented in Table 1.

**Table 1:** Item-Total Statistics

	Corrected Item-Total Correlation	Cronbach’s Alpha if Item Deleted
q1	.626	.928
q2	.613	.928
q3	.638	.928
q4	.714	.926
q5	.801	.923
q6	.724	.926
q7	.769	.924
q8	.476	.931
q9	.655	.927
q10	.549	.930
q11	.565	.930
q12	.723	.926
q13	.760	.925
q14	.597	.929
q15	.655	.927
q16	.672	.927
q17	.485	.933

### Construct validity

**Fig. 1:**
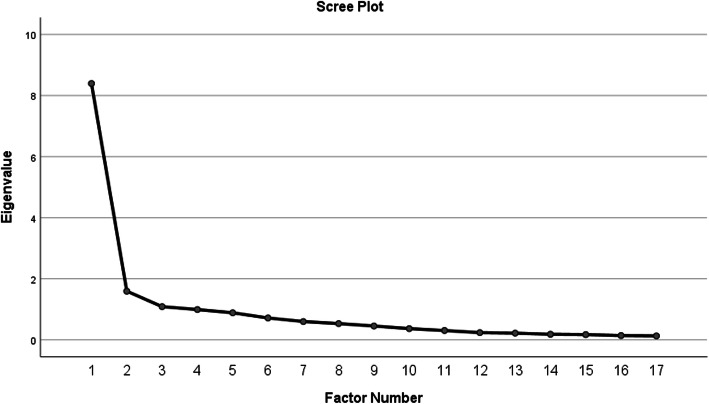
Scree plot

To determine the ability of the scale for factor analysis, KMO test was calculated (0.924). Bartlett’s test was also significant (X^2^ = 0.000). Besides, PROMAX rotation method was used to extract the factors and evaluate the construct validity. As a result, two factors with eigenvalues of more than 1 were extracted, which explained 58.47% of the total variance. The results are presented in Table 2 and Fig. 1.

**Table 2:** Exploratory Factor Analysis of the Farsi Version of the NSCTS

Factor	Items	Factor Loading	% of Variance	α	ICC
Factor1	q3	0.89	36.5	0.922	0.798
	q5	0.87			
	q12	0.82			
	q6	0.81			
	q4	0.80			
	q13	0.78			
	q7	0.73			
	q1	0.63			
	q2	0.61			
	q9	0.58			
	q16	0.49			
	q17	0.39			
Factor2	q11	0.97	21.96	0.799	0.862
	q10	0.92			
	q15	0.61			
	q8	0.56			
	q14	0.52			
Cumulative %			58.47	0.932	0.892

### Reliability

In this study, the scale has an overall alpha coefficient of 0.932. Cronbach’s alpha was 0.922 for the first factor, and 0.799 for the second factor. Also, in measuring test stability by test-retest method and using the interclass correlation coefficient (ICC) with 95% confidence interval, the ICC value was 0.892. The ICC of the first factor was 0.798 and the second factor was 0.862.

## Discussion

The results of this study showed that the NSCTS has good validity and reliability among Iranian nurses.

In the present study, the KMO value was 0.924. This rate was 0.943 in the study of Mamier and Taylor [6] and 0.85 in the study of Aslan et al. [8]. In exploratory factor analysis, two factors were identified by Promax rotation method. However, in the study of Aslan et al., and Mamier and Taylor, only one factor was identified. The eigenvalues in the study of Aslan et al. was 5.86 and explained 50.83% of the variance [8], and in the study of Mamier and Taylor, the factor explained 49.5% of the total variance [6]. Therefore, the findings of the present study are not in line with the above studies in terms of the number of factors obtained. It seems that the differences in studies in terms of the number of factors obtained are related to different rotation methods in factor analysis.

To assess the internal consistency of the tool, Cronbach’s reliability coefficient was measured (which was 0.932). In Aslan et al.’s study, Cronbach’s alpha coefficient was 0.86, and in Mamier and Taylor’s study, Cronbach’s alpha coefficient was 0.93; which this value is similar to Cronbach’s alpha coefficient obtained in this study [6, 8].

In Aslan et al.’s study in Turkey, the correlation of the total item was between 0.18 and 0.75 [8], and in Mamier and Taylor’s (2015) study, the correlation between items ranged from 0.40 to 0.80 [6]. However, in the present study, the correlation between items was from 0.4 to 0.9, which compared to previous studies, there was more correlation between items. A review of the literature indicates that the minimum acceptable value for correlation between items is 0.15. Therefore, the item-total correlation is also appropriate. In this case, similar to the study of Mamier and Taylor (2015), item number 17 has the lowest correlation (0.39). According to Mamier and Taylor, the reason might be found in the ambiguity and lack of understanding of the difference between care and spiritual care in nurses [6]. Another item that obtained the lowest correlation (0.56) in this study is item number 8 (Documented spiritual care you provided in a patient chart). Almost all participants answered “never” to this question. This means that if the nurse provides spiritual care to patients, there is no special place to record it. Systematic review of nursing reports has also shown that most nursing reports were incomplete, vague and incoherent and patients’ needs were not recorded [10]. Therefore, it is necessary to train nurses to record the actions taken by them, in addition to spiritual care and treatment.

Other tools have also been developed to measure spiritual care actions. These tools are similar to the NSCTS tool and their efficiency is determined in comparison with it. One of the scales in this field is the Spiritual Care Intervention-Provision (SCIP) scale, which was developed by Buchart et al. (2011) to assess nurses’ perceptions of their abilities to provide spiritual care services and the impact of this care on nurses’ spirituality. This scale was developed and reviewed in 2015 by Musa et al. in Jordan. In the factor analysis stage, two factors were identified that explained 42.88% of the variance. Cronbach’s alpha for scales and subscales (existential and religious dimensions) was 0.77 to 0.85. As a result, the study provided plausible evidence for the validity of SCIP among Jordanian Muslim nurses [5]. Therefore, it can be concluded from these studies (Musa et al., and the present study on Muslim nurses), that there is a relationship between nurses’ perceptions of their abilities to provide spiritual care services, and the reproducibility of these services and their frequency.

The Spiritual Care Intervention-Provision (SCIP) scale is designed to assess nurses’ spiritual care from their perspective. It has 17 items with three factor loadings that explain 57.33% of the variance. Reliability of scale and subscales was obtained above 0.8. The instrument’s psychometrics has been done among Western nurses. The four items on this scale indicate the number of spiritual care interventions; this number is too small to evaluate the satisfactory performance of spiritual care. The remaining 13 cases describe the nurses’ reaction to spiritual care. These cases do not assess a nurse’s willingness to provide spiritual nursing care, but rather measure the nurse’s performance after providing spiritual care. Thus, in comparison with NSCTS, this scale does not have the ability to assess the nurse’s satisfaction in providing spiritual care and spiritual care activities [3].

Palliative Care Spiritual Care Competency Scale (PCSCCS-M) is a self-reported scale developed by Chen et al. that potentially measures the competencies of students and palliative care professionals in providing spiritual care to patients. The validity and reliability of this scale was assessed by Yanli Hu et al. (2019) in China. The results of this study showed that PCSCCS-M is in an acceptable range in terms of validity, construct validity and internal correlation. In Hu’s study, as in the study of Chen et al., three factors were identified which Cronbach’s alpha was higher than 0.80 for all three factors. Therefore, it can be concluded that PCSCCS-M is a suitable tool for assessing the competence of spiritual care providers in China [11].

As can be seen from the literature review, the results of the validity and reliability of different tools in the field of measuring spiritual care in different societies with different cultures and religions are very similar to each other and are on appropriate scales. The reason for these similarities can be sought in the meaning of spiritual care, because spirituality is one of the basic needs of human life and is intertwined with the meaning and purpose of life [12]. Another important point is that spirituality plays an important part in most religions and even beyond any religion (11). But it is noteworthy that studies still report very little spiritual care by nurses; due to many obstacles such as inadequate education, uncertainty about spirituality and religion and the role of the nurse in dealing with them, fear, lack of time, and lack of privacy [13]. In this regard, nurses should be encouraged and provided with the necessary support.

The results of the present study and previous studies [8] showed that NSCT has the advantage of cross-cultural validity which can be used in different cultures and for nurses of all adult wards. Level of spiritual care performed by nurses can be measured with this scale and is an appropriate criteria for evaluating and educating nurses.

## Conclusion

According to the findings, the localized NSCTS tool in Iran shows a good validity and reliability and can be used in clinical wards for nurses. It can also be used for research, educational purposes and to improve the quality of spiritual care.

### Limitations

This study was conducted only with Muslim nurses in Iran. According to the majority of Muslims in the country, the results can be generalized to all Iranian nurses. However, it is recommended that this study be done in other religions as well. Also, other limitations of this study are the large number of incomplete and unanswered questionnaires and the small number of samples. It is recommended for future studies to use a larger sample.

## Data Availability

The datasets generated and/or analyzed during the current study are available from first and Corresponding author of this article upon reasonable request.
